# Difference in surgical outcomes of rectal cancer by study design: meta-analyses of randomized clinical trials, case-matched studies, and cohort studies

**DOI:** 10.1093/bjsopen/zraa067

**Published:** 2021-03-16

**Authors:** N Hoshino, T Sakamoto, K Hida, Y Takahashi, H Okada, K Obama, T Nakayama

**Affiliations:** 1 Department of Health Informatics, School of Public Health, Graduate School of Medicine, Kyoto University, Kyoto, Japan; 2 Department of Surgery, Kyoto University Graduate School of Medicine, Kyoto, Japan

## Abstract

**Background:**

RCTs are considered the standard in surgical research, whereas case-matched studies and propensity score matching studies are conducted as an alternative option. Both study designs have been used to investigate the potential superiority of robotic surgery over laparoscopic surgery for rectal cancer. However, no conclusion has been reached regarding whether there are differences in findings according to study design. This study aimed to examine similarities and differences in findings relating to robotic surgery for rectal cancer by study design.

**Methods:**

A comprehensive literature search was conducted using PubMed, Scopus, and Cochrane CENTRAL to identify RCTs, case-matched studies, and cohort studies that compared robotic *versus* laparoscopic surgery for rectal cancer. Primary outcomes were incidence of postoperative overall complications, incidence of anastomotic leakage, and postoperative mortality. Meta-analyses were performed for each study design using a random-effects model.

**Results:**

Fifty-nine articles were identified and reviewed. No differences were observed in incidence of anastomotic leakage, mortality, rate of positive circumferential resection margins, conversion rate, and duration of operation by study design. With respect to the incidence of postoperative overall complications and duration of hospital stay, the superiority of robotic surgery was most evident in cohort studies (risk ratio (RR) 0.83, 95 per cent c.i. 0.74 to 0.92, *P *<* *0.001; mean difference (MD) –1.11 (95 per cent c.i. –1.86 to –0.36) days, *P *=* *0.004; respectively), and least evident in RCTs (RR 1.12, 0.91 to 1.38, *P *=* *0.27; MD –0.28 (–1.44 to 0.88) days, *P *=* *0.64; respectively).

**Conclusion:**

Results of case-matched studies were often similar to those of RCTs in terms of outcomes of robotic surgery for rectal cancer. However, case-matched studies occasionally overestimated the effects of interventions compared with RCTs.

## Teaser

The incidence of postoperative overall complications did not differ significantly between robotic surgery and laparoscopic surgery in RCTs and case-matched studies, whereas significant differences were observed in cohort studies. The superiority of robotic surgery was most evident in cohort studies, least evident in RCTs, and intermediate (between cohort studies and RCTs) in case-matched studies.

meta analysis on rectal cancer surgery

## Introduction

RCTs are currently considered the standard for studying treatment effects in surgical research^1^^,^^2^. However, RCTs require considerable resources such as time, resources, costs, and collaboration among various specialists to ensure patient security, standardization of interventions, and data correctness. Although blinding is an important design feature of RCTs, blinding of outcome assessors, as well as for patients and surgeons, is difficult to achieve in surgical research, making it difficult to conduct high-quality RCTs^3^^,^^4^. Moreover, it is often impossible to conduct surgical RCTs for various reasons, such as feasibility and ethics^1^. Thus, findings from high-quality RCTs are not always available in surgical research^5^.

Recently, matching methods such as propensity score matching have been adopted as alternative methods to randomization. A number of studies using matching methods have been published, and such studies are generally referred to as case-matched studies^1^^,^^6–8^. However, only measurable confounding factors can be adjusted for in case-matched studies, and reports of such studies occasionally lack sufficient details of matching variables and patient characteristics^9–12^.

Both high- and low-quality RCTs and case-matched studies have been published. Apart from methodological differences between the two types of study, such as patient selection and adjustment for confounders, it remains unclear whether there are differences in results by study design^2^^,^^13^.

RCTs and case-matched studies have been conducted to examine the potential superiority of robotic surgery over laparoscopic surgery for rectal cancer, a topic of major interest among surgeons. However, no conclusion has been reached regarding whether differences exist by study design. On this basis, the present study aimed to examine similarities and differences in findings related to surgical outcomes for rectal cancer according to study design.

## Methods

Eligible studies were those comparing robotic *versus* laparoscopic surgery for rectal cancer. Studies of transanal surgery were excluded. RCTs, case-matched studies, and cohort studies were subjected to analysis. Both prospective and retrospective studies were included in non-RCT studies. No restrictions were placed regarding methods of randomization or matching.

A comprehensive literature search was conducted on 12 June 2019 using PubMed, Scopus, and the Cochrane Central Register of Controlled Trials (CENTRAL). The following search terms were used: ‘rectal cancer’, ‘surgery’, ‘robot’, ‘laparoscopy’, and related terms (*Appendix S1*). Duplications were excluded by checking author names, year of publication, and study characteristics (such as study design, setting, and period). Two authors independently screened the extracted publications according to title and abstract, and then reviewed the full text of potentially eligible articles. Disagreement was resolved by discussion.

Data extracted included: study design and setting, number and characteristics of patients, type of surgery, and short-term surgical outcomes. The extracted data were checked for consistency, and discordance was resolved by discussion. For cohort studies, unadjusted data were extracted.

### Outcome measures

Primary outcomes were: incidence of postoperative overall complications, incidence of anastomotic leakage, and mortality. Secondary outcomes were: duration of hospital stay, conversion rate, duration of operation, estimated blood loss, rate of positive circumferential resection margins, and quality of total mesorectal excision.

### Statistical analysis

Data synthesis was performed using Review Manager 5.3 (The Nordic Cochrane Centre, Copenhagen, Denmark). A random-effects model was used for all meta-analyses, as all types of rectal cancer surgery were included in the present review. An inverse-variance method was used for continuous variables, and the Mantel–Haenszel method for dichotomous variables. Mean difference (MD) with 95 per cent confidence interval was used for continuous variables when a single measure was included in the meta-analysis. Median (range) values were converted to mean(s.d.)^14^. Risk ratio (RR) with 95 per cent c.i. was used for dichotomous variables. When an outcome was rare, risk difference (RD) was used instead of RR. *P *<* *0.050 (2-sided) was considered statistically significant.

## Results

**Fig. 1:**
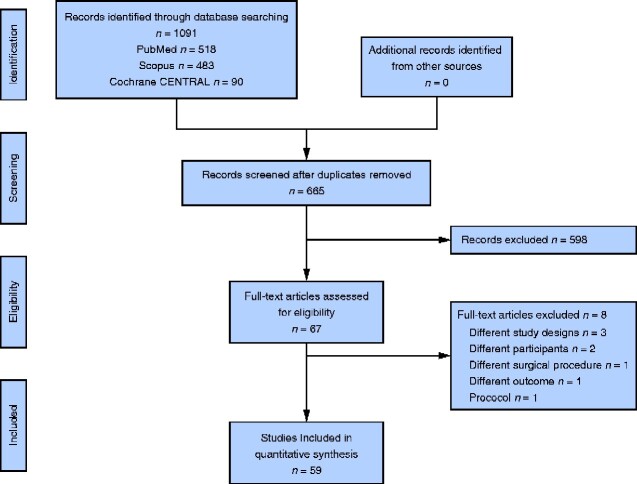
Flow diagram showing selection of studies for review CENTRAL, Central Register of Controlled Trials.

The literature search yielded 1091 articles in total. Among these, 426 duplicates were removed, and the remaining 665 articles were screened for eligibility based on title and abstract. After screening, 67 articles were subjected to full-text review, and 59 articles that met the inclusion criteria were included in the present review (*Fig. 1*)^15–73^. Reviewed studies included seven RCTs, 13 case-matched studies, and 39 cohort studies; two were conducted internationally, and 57 were reported from 17 countries. All case-matched studies were retrospective. Among the 13 case-matched studies, propensity score matching was used in seven, and manual matching in one; no matching method was described in five. Variables used for matching included patient age, sex, co-morbidity, tumour location and stage, and surgical procedure. Among cohort studies, one was prospective and 38 were retrospective (*Table 1*).

**Table 1:** Patient characteristics

Reference	Setting		Study interval	Study type	Surgical procedures	No. of patients	
	Country	Institution				Robotic	Laparoscopic
**RCTs**							
Baik *et al.*^15^	Korea	Single	Apr 2006 to Feb 2007	Prospective	LAR	18	18
Debakey *et al.*^16^	Egypt	Single	April 2015 to Feb 2017	Prospective	AR, LAR, APR	21	24
Jayne *et al.*^17^	International	Multiple	Jan 2011 to Sept 2014	Prospective	AR, LAR, APR	236	230
Kim *et al.*^18^	Korea	Single	Feb 2012 to Mar 2015	Prospective	LAR, HO, APR	66	73
Patriti *et al.*^19^	Italy	Single	Mar 2004 to Oct 2008	Prospective	PME, TME, APR, CAA	29	37
Tolstrup *et al.*^20^	Denmark	Single	Nov 2012 to Apri 2014	Prospective	PME, TME, APR, ISR	25	26
Wang *et al.*^21^	China	Single	Nov 2010 to Sept 2013	Prospective	LAR, HO	71	66
**Case-matched studies**							
Ackerman *et al.*^22^	USA	Multiple	Jan 2012 to Dec 2014	Retrospective	AR	533	533
Allemann *et al.*^23^	Switzerland	Single	May 2012 to Jan 2014	Retrospective	LAR, APR, ISR	20	40
Baek *et al.*^24^	Korea	Single	Apr 2003 to Mar 2009	Retrospective	LAR, CAA, APR	41	41
Cho *et al.*^25^	Korea	Single	Jan 2007 to Jun 2011	Retrospective	LAR, CAA	278	278
Kim *et al.*^26^	Korea	Single	Mar 2010 to Jan 2012	Retrospective	LAR, HO, APR	33	66
Kim *et al.*^27^	Korea	Single	Apr 2007 to Mar 2014	Retrospective	AR, LAR, ISR, APR	224	224
Kim *et al.*^28^	Korea	Single	2009–2013	Retrospective	LAR, CAA, APR	130	130
Koh *et al.*^29^	Singapore	Single	Aug 2008 to Aug 2011	Retrospective	LAR, APR	19	19
Panteleimonitis *et al.*^30^	International	Multiple	2006–2012	Retrospective	AR, LAR, HO, APR	63	61
Park *et al.*^31^	Korea	Single	Dec 2005 to Jun 2009	Retrospective	LAR, CAA, APR	41	82
Park *et al.*^32^	Korea	Single	Feb 2009 to Dec 2010	Retrospective	LAR, ISR, APR	32	32
Park *et al.*^33^	Korea	Multiple	Jan 2008 to May 2011	Retrospective	ISR	106	106
Sugoor *et al.*^34^	India	Single	Jun 2013 to Dec 2017	Retrospective	AR, LAR, ISR, TPE	84	84
**Cohort studies**							
Ahmed *et al.*^35^	UK	Single	May 2013 to Nov2015	Retrospective	AR, APR, HO, TPC	99	88
Aselmann *et al.*^36^	Germany	Single	Jan 2011 to Dec 2016	Retrospective	LAR	44	41
Baek *et al.*^37^	Korea	Single	Jan 2007 to Dec 2010	Retrospective	LAR, CAA	47	37
Baik *et al.*^38^	Korea	Single	Apr 2006 to Sep 2007	Prospective	LAR	56	57
Bedirli *et al.*^39^	Turkey	Single	Jan 2013 to Jun 2015	Retrospective	LAR	35	28
Bianchi *et al.*^40^	Italy	Single	Mar 2008 to Jun 2009	Retrospective	AR, APR	25	25
Bo *et al.*^41^	China	Single	Mar 2010 to Jun 2016	Retrospective	AR, LAR, ISR, APR, HO	556	1139
Crolla *et al.*^42^	Netherlands	Single	2005–2015	Retrospective	LAR, HO, APR	168	184
D’Annibale *et al.*^43^	Italy	Single	2004–2012	Retrospective	TME	50	50
Erguner *et al.*^44^	Turkey	Single	Feb 2008 to Jun 2011	Retrospective	LAR	27	37
Esen *et al.*^45^	Turkey	Single	Dec 2014 to Aug 2017	Retrospective	TME, PME	100	78
Fernandez *et al.*^46^	USA	Single	2002–2012	Retrospective	LAR, APR	13	59
Feroci *et al.*^47^	Italy	Multiple	Jan 2008 to Dec 2014	Retrospective	TME	53	58
Gorgun *et al.*^48^	USA	Single	Jan 2011 to Jun 2014	Retrospective	AR, APR, CAA	29	27
Huang *et al.*^49^	Taiwan	Single	Jan 2012 to Apr 2015	Retrospective	LAR, ISR	40	38
Ielpo *et al.*^50^	Spain	Single	Oct 2010 to Jul 2013	Retrospective	LAR, APR	56	87
Ielpo *et al.*^51^	Spain	Single	Oct 2010 to Mar 2017	Retrospective	LAR, APR, CAA	86	112
Kamali *et al.*^52^	UK	Single	Jul 2014 to Sep 2016	Retrospective	AR	18	18
Kamali *et al.*^53^	UK	Single	Feb 2015 to Aug 2016	Retrospective	APR	11	11
Kim *et al.*^54^	Korea	Single	Jun 2009 to Nov 2009	Retrospective	SSP, HO	30	39
Kim *et al.*^55^	Korea	Single	May 2006 to Dec 2014	Retrospective	LAR, ISR, APR	50	35
Kuo *et al.*^56^	Taiwan	Single	Nov 2009 to Jul 2013	Retrospective	ISR	36	28
Law *et al.*^57^	China	Single	Jan 2008 to Jun 2015	Retrospective	LAR, HO, APR	220	171
Levic *et al.*^58^	Denmark	Multiple	2010–2012	Retrospective	LAR, HO, APR	56	36
Lim *et al.*^59^	Korea	Single	Jan 2006 to Dec 2010	Retrospective	LAR, ISR, CAA, APR	74	64
Liu *et al.*^60^	China	Single	Jul 2015 to Oct 2017	Retrospective	AR, APR	80	116
Megevand *et al.*^61^	Italy	Single	Jan 2011 to Dec 2015	Retrospective	AR, HO, APR	35	35
Panteleimonitis *et al.*^62^	UK	Single	Dec 2006 to Sep 2014	Retrospective	AR, HO, APR	48	78
Park *et al.*^63^	Korea	Single	Mar 2008 to Jul 2011	Retrospective	ISR	40	40
Park *et al.*^64^	Korea	Single	Apr 2006 to Aug 2011	Retrospective	LAR	133	84
Pigazzi *et al.*^65^	USA	Single	Sep 2004 to Oct 2005	Retrospective	LAR	6	6
Popescu *et al.*^66^	Romania	Single	1995–2010	Retrospective	AR, APR	38	84
Saklani *et al.*^67^	Korea	Single	Jan 2006 to Dec 2010	Retrospective	LAR, CAA, ISR, APR	74	64
Serin *et al.*^68^	Turkey	Single	Jan 2005 to Dec 2013	Retrospective	LAR, ISR	14	65
Shin *et al.*^69^	Korea	Single	Jan 2011 to Dec 2014	Retrospective	ISR	34	60
Tam *et al.*^70^	USA	Single	Feb 2011 to Feb 2013	Retrospective	AR, LAR, ISR, APR, TPC	21	21
Yamaguchi *et al.*^71^	Japan	Single	Apr 2010 to Apr 2015	Retrospective	LAR, ISR, HO, APR	203	239
Yoo *et al.*^72^	Korea	Single	Sep 2006 to Aug 2008	Retrospective	ISR	44	26
Yoon *et al.*^73^	Korea	Single	Jun 2006 to Dec 2010	Retrospective	AR, LAR	17	61

LAR, low anterior resection; AR, anterior resection; APR, abdominoperineal resection; HO, Hartmann’s operation; PME, partial mesorectal excision; TME, total mesorectal excision; CAA, coloanal anastomosis; ISR, intersphincteric resection; TPE, total pelvic excision; TPC, total proctocolectomy; SSP, sphincter-saving procedure.

### Incidence of postoperative overall complications

**Fig. 2:**
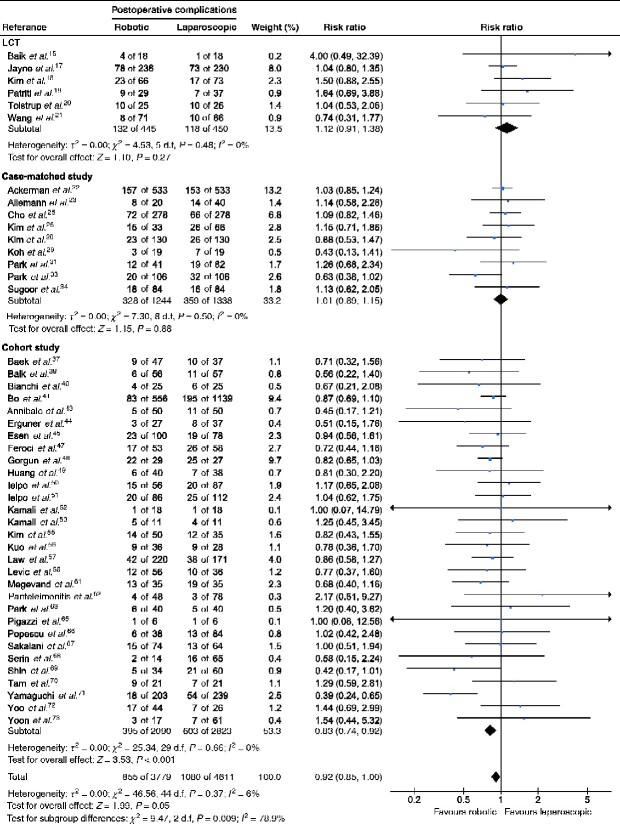
Results of meta-analysis stratified by study design: incidence of postoperative overall complications A Mantel–Haenszel random-effects model was used for statistical analysis. Mean differences are shown with 95 per cent confidence intervals.

Forty-five studies involving a total of 8390 patients (6 RCTs, 895 patients; 9 case-matched studies, 2582 patients; 30 cohort studies, 4913 patients) reported on the incidence of overall complications and were included in a meta-analysis stratified by study design. The incidence of overall complications did not differ significantly between robotic and laparoscopic surgery in RCTs (RR 1.12, 95 per cent c.i. 0.91 to 1.38; *P *=* *0.27) and case-matched studies (RR 1.01, 0.89 to 1.15; *P *=* *0.88). In cohort studies, however, robotic surgery was associated with a significantly lower incidence of overall postoperative complications compared with laparoscopic surgery (RR 0.83, 0.74 to 0.92; *P *<* *0.001) (*Table 2* and *Fig. 2*).

**Table 2:** Summary of meta-analyses by study design

	Measure	RCTs			Case–matched studies			Cohort studies		
		No. of studies	No. of patients	Point estimate	No. of studies	No. of patients	Point estimate	No. of studies	No. of patients	Point estimate
**Primary outcomes**										
Postoperative overall complications	RR	6	895	1.12 (0.91,1.38)	9	2582	1.01 (0.89, 1.15)	30	4913	0.83 (0.74, 0.92)
Anastomotic leakage	RR	6	784	0.97 (0.67, 1.39)	12	2222	0.97 (0.74, 1.29)	35	5366	0.94 (0.74, 1.18)
Mortality	RD	6	904	–0.00 (–0.01, 0.01)	10	1910	–0.00 (–0.01, 0.00)	26	5025	–0.00 (–0.00, 0.00)
**Secondary outcomes**										
Duration of hospital stay (days)	MD	6	781	–0.28 (–1.44, 0.88)	8	1904	–0.59 (–1.18, 0.00)	25	4966	–1.11 (–1.86, –0.36)
Conversion rate	RR	6	803	0.42 (0.17, 1.03)	11	2976	0.40 (0.31, 0.51)	36	6034	0.34 (0.24, 0.49)
Duration of operation (min)	MD	6	803	33.53 (–3.25, 70.31)	7	1644	83.41 (54.37, 112.45)	29	5345	44.70 (32.40, 57.00)
Estimated blood loss (ml)	MD	3	250	36.09 (–136.41, 208.59)	5	1095	–16.23 (–69.27, 36.82)	21	4438	–13.49 (–29.11, 2.14)
Positive circumferential resection margins	RR	3	664	0.88 (0.46, 1.69)	10	2046	1.05 (0.70, 1.57)	29	5545	0.84 (0.63, 1.12)
Quality of total mesorectal excision	RR	4	686	1.08 (0.95, 1.23)	2	133	1.34 (0.74, 2.42)	9	1585	1.14 (1.05, 1.23)

Values in parentheses are 95 per cent confidence intervals. RR, risk ratio; RD, risk difference; MD, mean difference.

### Incidence of anastomotic leakage

Fifty-three studies involving a total of 8372 patients (6 RCTs, 784 patients; 12 case-matched studies, 2222 patients; 35 cohort studies, 5366 patients) that reported on the incidence of anastomotic leakage were included in a meta-analysis stratified by study design. The incidence of anastomotic leakage did not differ significantly between robotic and laparoscopic surgery in RCTs (RR 0.97, 95 per cent c.i. 0.67 to 1.39; *P *=* *0.86), case-matched studies (RR: 0.97, 0.74 to 1.29; *P *=* *0.85), and cohort studies (RR0.94, 0.74 to 1.18; *P *=* *0.57) (*Table 2* and *Fig. S1*).

### Mortality

Forty-two studies involving a total of 7839 patients (6 RCTs, 904 patients; 10 case-matched studies, 1910 patients; 26 cohort studies, 5025 patients) that reported on mortality were included in a meta-analysis stratified by study design. Mortality did not differ significantly between robotic and laparoscopic surgery in RCTs (RD –0.00, 95 per cent c.i. –0.01 to 0.01; *P *=* *0.99), case-matched studies (RD –0.00, –0.01 to 0.00; *P *=* *0.38), and cohort studies (RD –0.00, –0.00 to 0.00; *P *=* *0.45) (*Table 2* and *Fig. S2*).

### Duration of hospital stay

**Fig. 3:**
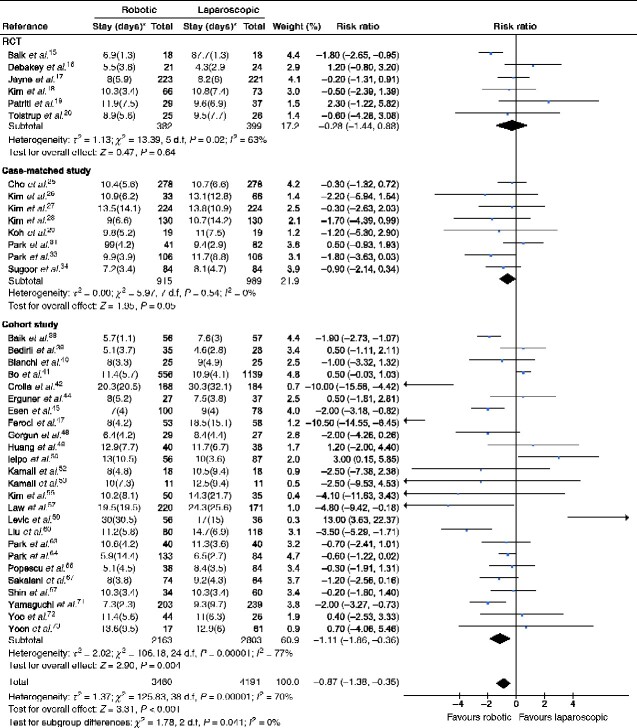
Results of meta-analysis stratified by study design: duration of hospital stay An inverse-variance random-effects model was used for statistical analysis. Mean differences are shown with 95 per cent confidence intervals. *Values are mean(s.d.).

Thirty-nine studies involving a total of 7651 patients (6 RCTs, 781 patients; 8 case-matched studies, 1904 patients; 25 cohort studies, 4966 patients) that reported on duration of hospital stay were included in a meta-analysis stratified by study design. Duration of hospital stay did not differ significantly between robotic and laparoscopic surgery in RCTs (MD –0.28 (95 per cent c.i. –1.44 to 0.88) days; *P *=* *0.64) and case-matched studies (MD –0.59 (–1.18 to 0.00) days; *P *=* *0.05). In cohort studies, however, robotic surgery was associated with a significantly shorter hospital stay than laparoscopic surgery (MD –1.11 (–1.86 to –0.36) days; *P *=* *0.004) (*Table 2* and *Fig. 3*).

### Conversion rate

Fifty-three studies involving a total of 9813 patients (6 RCTs, 803 patients; 11 case-matched studies, 2976 patients; 36 cohort studies, 6034 patients) that reported on conversion rate were included in a meta-analysis stratified by study design. Conversion rate did not differ significantly between robotic and laparoscopic surgery in RCTs (RR 0.42, 95 per cent c.i. 0.17 to 1.03; *P *=* *0.06). On the other hand, robotic surgery was associated with a significantly lower conversion rate than laparoscopic surgery in case-matched studies (RR 0.40, 0.31 to 0.51; *P *<* *0.001) and cohort studies (RR 0.34, 0.24 to 0.49; *P *<* *0.001) (*Table 2* and *Fig. S3*).

### Duration of operation

Forty-two studies involving a total of 7792 patients (six RCTs, 803 patients; seven case-matched studies, 1644 patients; 29 cohort studies, 5345 patients) that reported on duration of operation were included in a meta-analysis stratified by study design. Duration of operation did not differ significantly between robotic and laparoscopic surgery in RCTs (MD 33.53 (95 per cent c.i. –3.25 to 70.31) min; *P *=* *0.07). However, robotic surgery was associated with a significantly longer operating time than laparoscopic surgery in case-matched studies (MD 83.41 (54.37 to 112.45) min; *P *<* *0.001) and cohort studies (MD 44.70 (32.40 to 57.00) min; *P *<* *0.001) (*Table 2* and *Fig. S4*).

### Estimated blood loss

Twenty-nine studies involving a total of 5783 patients (3 RCTs, 250 patients; 5 case-matched studies, 1095 patients; 21 cohort studies, 4438 patients) that reported on estimated blood loss were included in a meta-analysis stratified by study design. Estimated blood loss did not differ significantly between robotic and laparoscopic surgery in RCTs (MD 36.09 (95 per cent c.i. –136.41 to 208.59) ml; *P *=* *0.68), case-matched studies (MD –16.23 (–69.27 to 36.82) ml; *P *=* *0.55) and cohort studies (MD –13.49 (–29.11 to 2.14) ml; *P *=* *0.09) (*Table 2* and *Fig. S5*).

### Rate of positive circumferential resection margins

Forty-two studies involving a total of 8255 patients (3 RCTs, 664 patients; 10 case-matched studies, 2046 patients; 29 cohort studies, 5545 patients) that reported on the rate of positive circumferential resection margins were included in a meta-analysis stratified by study design. The rate of positive circumferential resection margins did not differ significantly between robotic and laparoscopic surgery in RCTs (RR 0.88, 95 per cent c.i. 0.46 to 1.69; *P *=* *0.70), case-matched studies (RR 1.05, 0.70 to 1.57; *P *=* *0.81) and cohort studies (RR 0.84, 0.63 to 1.12; *P *=* *0.23) (*Table 2* and *Fig. S6*).

### Quality of total mesorectal excision

Fifteen studies involving a total of 1585 patients (4 RCTs, 686 patients; 2 case-matched studies, 133 patients; 9 cohort studies, 1585 patients) that reported on the quality of total mesorectal excision were included in a meta-analysis stratified by study design. The quality of total mesorectal excision did not differ significantly between robotic and laparoscopic surgery in RCTs (RR 1.08, 95 per cent c.i. 0.95 to 1.23; *P *=* *0.22) and case-matched studies (RR 1.34, 0.74 to 2.42; *P *=* *0.33). In cohort studies, however, robotic surgery was associated with a significantly higher quality of total mesorectal excision than laparoscopic surgery (RR 1.14, 1.01 to 1.28; *P *=* *0.03) (*Table 2* and *Fig. S7*).

## Discussion

The present systematic review and meta-analyses revealed that, among 59 studies that compared robotic *versus* laparoscopic surgery for rectal cancer, similarities and differences in findings were observed by study design, particularly between RCTs and case-matched studies. Among the nine outcomes assessed, two (estimated blood loss and quality of total mesorectal excision) were difficult to compare by meta-analyses, as the number of included studies was small and the 95 per cent confidence intervals were wide.

With respect to the incidence of anastomotic leakage, mortality, and rate of positive circumferential resection margins, meta-analyses for each study design revealed no significant differences between robotic and laparoscopic surgery, suggesting that findings related to these outcomes did not differ by study design. On the other hand, meta-analyses of case-matched studies and cohort studies, but not RCTs, revealed significant differences between robotic and laparoscopic surgery with respect to conversion rate and duration of operation. However, the number of included patients was lower for RCTs than for case-matched studies and cohort studies, and 95 per cent confidence intervals were also wider, suggesting that the statistical power might have been lower. Given the wide range of 95 per cent confidence intervals and lower statistical power, the difference between the three study designs in terms of conversion rate and operating time in the meta-analysis could be considered minimal.

The incidence of postoperative overall complications (primary outcome) and duration of hospital stay (secondary outcome) did not differ significantly between robotic surgery and laparoscopic surgery in RCTs and case-matched studies, whereas significant differences were observed in cohort studies. In-depth analyses of the distribution of 95 per cent confidence across study designs showed that outcomes from case-matched studies fell between those of RCTs and cohort studies in meta-analyses. Specifically, superiority of robotic surgery was most evident in cohort studies, least evident in RCTs, and intermediate (between cohort studies and RCTs) in case-matched studies. These differences by study design might reflect the degree of adjustment for confounding factors between study designs. All confounding factors including measurable and unmeasurable factors could be adjusted for in RCTs, whereas confounding factors in cohort studies were not controlled for in the present meta-analyses because the data were unadjusted.

In this review, the results of meta-analyses did not show differences in most of the outcomes assessed. This is consistent with a previous report^2^ that results of RCTs were similar to those of case-matched studies in cardiac surgery. On the other hand, other authors^13^ reported that case-matched studies tended to overestimate the efficacy of interventions compared with RCTs in patients with acute coronary syndrome. In the present review, the incidence of postoperative overall complications differed by study design, whereas that of anastomotic leakage did not. Postoperative overall complications include anastomotic leakage and so the rates are higher for postoperative overall complications than for anastomotic leakage. Because the statistical power was greater for postoperative overall complications than for anastomotic leakage, the difference in power might have had some influence. Moreover, although anastomotic leakage can be assessed objectively, other complications such as surgical-site infection and ileus are often influenced by subjective judgements. Duration of hospital stay can also be influenced by subjective judgements because the timing of discharge may depend on surgeon preference. In addition, experimental and comparator interventions are usually performed during the same interval in RCTs, whereas historical comparators are sometimes used in cohort studies. Duration of hospital stay tends to shorten as time progresses owing to the introduction of newer and more effective treatment modalities. In this regard, robotic surgery is a newer technique than laparoscopic surgery. Thus, hospital stay after robotic surgery might be shorter in RCTs than in cohort studies. Clinicians should interpret findings related to these outcomes with caution, and consider the study design when doing so.

The strength of the present review is the large number of studies examined. In total, 59 studies were reviewed, compared with 5–23 in previous systematic reviews^74^. Moreover, previous studies that focused on differences by study design often investigated a single outcome for each comparison^5^^,^^75^, whereas nine outcomes for a single comparison (robotic *versus* laparoscopic surgery) were investigated here to highlight differences in surgical outcomes. However, this study also has some limitations. The numbers of studies and patients differed among the three types of study, and tended to be lower in RCTs. The present review included only published data and did not consider the quality of each study.

Finally, the results of case-matched studies were often similar to those of RCTs with respect to objective outcomes of robotic surgery for rectal cancer. However, case-matched studies potentially overestimated the effect of interventions compared with RCTs in terms of subjective outcomes.

## Funding

The study received no funding.

## Supplementary Material

zraa067_Supplementary_Data
