# Supplementary material for: Difference in surgical outcomes of rectal cancer by study design: meta-analyses of randomized clinical trials, case-matched studies, and cohort studies
Source: BJS Open. 2021 Mar 16;5(2):zraa067. doi: 10.1093/bjsopen/zraa067 (PMC7962725; doi:10.1093/bjsopen/zraa067)
Supplement: zraa067_Supplementary_Data [file zraa067_supplementary_data.zip › Appendix_1.docx]

**Appendix 1: Search strategy for PubMed**

#1 "Rectal Neoplasms"[MeSH Terms]

#2 rectum[Tiab] OR rectal[Tiab]

#3 neoplasm[Tiab] OR cancer[Tiab] OR tumor[Tiab] OR carcinoma[Tiab]

#4 #2 AND #3

#5 #1 OR #4

#6 Colectomy[MeSH Terms]

#7 surgery[Tiab] OR operation[Tiab] OR resection[Tiab] OR proctectomy[Tiab] OR “anterior resection”[Tiab] OR Miles[Tiab] OR “abdominoperineal resection”[Tiab] OR Hartmann[Tiab] OR proctocolectomy[Tiab] OR “total mesorectum excision”[Tiab] OR “total mesorectal excision”[Tiab]

#8 #6 OR #7

#9 “Robotic Surgical Procedures”[MeSH Terms]

#10 robot[Tiab] OR robotic[Tiab] OR “da vinci”[Tiab]

#11 #9 OR #10

#12 Laparoscopy[MeSH Terms]

#13 laparoscopy[Tiab] OR laparoscopic[Tiab]

#14 #12 OR #13

#15 #5 AND #8 AND #11 AND #14
