# Supplementary material for: Difference in surgical outcomes of rectal cancer by study design: meta-analyses of randomized clinical trials, case-matched studies, and cohort studies
Source: BJS Open. 2021 Mar 16;5(2):zraa067. doi: 10.1093/bjsopen/zraa067 (PMC7962725; doi:10.1093/bjsopen/zraa067)
Supplement: zraa067_Supplementary_Data [file zraa067_supplementary_data.zip › Suppl Fig legends.docx]

Fig.S1 Results of meta-analysis stratified by study design: incidence of anastomotic leakage

Fig.S2 Results of meta-analysis stratified by study design: mortality

Fig.S3 Results of meta-analysis stratified by study design: conversion rate

Fig.S4 Results of meta-analysis stratified by study design: operating time

Fig.S5 Results of meta-analysis stratified by study design: estimated blood loss

Fig.S6 Results of meta-analysis stratified by study design: rate of positive circumferential resection margins

Fig.S7 Results of meta-analysis stratified by study design: quality of total mesorectal excision
